# Editorial: Novel insights into the comorbidities and mortality in patients with diabetes

**DOI:** 10.3389/fendo.2024.1406131

**Published:** 2024-04-10

**Authors:** Sen Li, Haiqiang Dou, Ping Wang, Hongcai Shang

**Affiliations:** ^1^ School of Life Sciences, Beijing University of Chinese Medicine, Beijing, China; ^2^ Department of Physiology, Sahlgrenska Academy, University of Gothenburg, Gothenburg, Sweden; ^3^ Precision Health Program, Michigan State University, East Lansing, MI, United States; ^4^ Department of Radiology, College of Human Medicine, Michigan State University, East Lansing, MI, United States; ^5^ Key Laboratory of Chinese Internal Medicine of Ministry of Education and Beijing, Beijing University of Chinese Medicine Affiliated Dongzhimen Hospital, Beijing, China

**Keywords:** diabetes, diabetes complications, risk factors, comorbidities, mortality

Diabetes is a persistent metabolic condition characterized by elevated blood sugar levels, leading to various complications. Around 537 million individuals globally were reported to have diabetes in 2021, and this number continues to rise (1). Diabetes and its associated complications substantially diminish the quality of life for patients and contribute to over 4 million deaths annually. Diabetes and its complications not only elevate premature mortality rates but also amplify the economic burden on society. Multiple studies indicate that diabetes can lead to chronic multisystem damage and dysfunction (2, 3). Moreover, diabetes frequently coincides with other illnesses that may share certain risk factors. For instance, individuals with diabetes have a higher likelihood of also experiencing hypertension, chronic obstructive pulmonary disease, or asthma (4, 5). Nevertheless, there remains a gap in understanding the correlation between diabetes and other potential comorbidities such as hepatitis infection and periodontal disease (PD). Moreover, the common risk factors that contribute to the development of diabetes and its comorbidities are not yet fully understood. Various comorbidities, such as cardiovascular and cerebrovascular diseases, significantly increase the risk of mortality in diabetes patients. However, the relationship between diabetes and the mortality rates of other diseases, like endometrial or kidney cancer, shows inconsistency. Additional research is required to uncover the heightened mortality linked with comorbidities in diabetic individuals.

This current Research Topic comprises a total of 12 studies. These studies specifically assess the prevalence and risk factors associated with diabetes complications, while also examining potential biological indicators linked to diabetes-related complications.

As commonly acknowledged, managing blood glucose levels serves as an efficient method for averting vascular complications in diabetes. Inadequate long-term management of blood sugar levels can lead to damage in nerves and blood vessels across the body, including those in the feet and eyes. In a review article, Waibel et al. examined research findings concerning the management, morbidity, mortality, and associated costs related to diabetic foot disorder. In a cohort study reported by Zang et al., encompassing 800 participants, it was revealed that 71.2% of patients diagnosed with vision-threatening diabetic retinopathy (VTDR) were oblivious to their condition. These results underscore the insufficient screening endeavors for diabetic retinopathy (DR) within their study population. In a retrospective comparative investigation undertaken by Yan et al., they examined 2,961 individuals diagnosed with type 2 diabetes (T2D) to assess the occurrence rates of DR and VTDR categorized according to the duration of diabetes. The research revealed that while the likelihood of developing DR is relatively lower among individuals with ≤3 years’ duration of T2D, the potential for VTDR should not be overlooked, particularly in patients with elevated glycated hemoglobin levels and/or diabetic nephropathy. Therefore, there is a need for more frequent retinal screening in individuals newly diagnosed with diabetes.

People with diabetes face a heightened risk of developing and experiencing PD. In a cross-sectional investigation, Gregorczyk-Maga et al. collected gingival crevicular fluid samples from adults with type 1 diabetes (T1D) undergoing continuous subcutaneous insulin infusion therapy and compared them with non-diabetic controls. They conducted a metagenomic/metabolomic analysis, leading to the conclusion that despite maintaining good metabolic control of diabetes, individuals with T1D remain vulnerable to the onset of PD. Additionally, Serón et al. undertook an extensive review of literature spanning from 2000 to 2023, examining the pathophysiological connections between periodontitis, diabetes, and atherosclerotic cardiovascular diseases. Their analysis indicates that integrating regular screening and treatment for periodontitis into national health programs is a cost-effective strategy to enhance metabolic management, decrease complications, and improve the overall well-being of individuals with diabetes.

It’s crucial to comprehend and steer clear of the typical risk factors linked to the development and complications of diabetes for a favorable prognosis. In a systematic review and meta-analysis undertaken by Li et al., a cumulative total of 60 studies encompassing 5,960,224 participants were incorporated. The research findings indicated that hypoglycemia is correlated with an elevated risk of cardiac arrhythmia and mortality. Yang et al. performed a retrospective examination of 3,291 septic patients with diabetes from the extensive real-world database of the Medical Information Mart for Intensive Care. They pinpointed ten clinical variables, including respiratory failure, as prognostic factors for predicting the 28-day all-cause mortality in septic patients with diabetes. Zhao et al. utilized a Mendelian randomization (MR) approach to explore shared risk factors among T1D, T2D, and chronic kidney disease (CKD). They discovered that the eosinophil percentage may serve as a common risk factor for both T1D and CKD. Additionally, they identified various traits, including obesity and blood lipids, as shared risk factors for T2D and CKD. In another MR investigation carried out by Zhao et al., differing causal relationships were observed between T2D and chronic liver diseases in East Asians and Europeans.

Examining potential biological indicator in diabetic patients can assist in the early detection of diabetes complications, thereby enabling prompt intervention and treatment. Jin et al. performed a systematic review and meta-analysis of 42 studies, identifying a total of 68 serum/plasma biomarkers. They concluded that lipid metabolism biomarkers were the most reported factors to be associated with the risk of cardiometabolic multimorbidity. Su et al. studied 675 patients diagnosed with T2D who underwent percutaneous coronary intervention. Their findings suggested that fibrinogen levels have the potential to serve as a noninvasive biomarker for predicting coronary anatomical complexity in individuals with T2D, aiding in the early detection of those at elevated risk. In a cohort investigation reported by Ryu et al., which included 30,164 Korean individuals aged over 60 years, it was discovered that the metabolic score for insulin resistance index could serve as a valuable predictive indicator for all-cause mortality and cancer mortality within their study group. However, it did not exhibit predictive capability for cardiovascular mortality.

The studies encompassed within this Research Topic delve into the shared risk factors and potential biological markers associated with complications in diabetes. These findings carry substantial implications for the diagnosis, prevention, and treatment of diabetic complications. Through comprehension of these factors and biomarkers, there arises the opportunity to enhance the management of health conditions in diabetic patients and actively contribute to mitigating the risk of complications.

## Author contributions

SL: Writing – original draft. HD: Writing – review & editing. PW: Writing – review & editing. HS: Writing – review & editing.
